# Evidence that Nitric Oxide is Involved in the Blood Pressure Lowering Effect of the Peptide AVFQHNCQE in Spontaneously Hypertensive Rats

**DOI:** 10.3390/nu11020225

**Published:** 2019-01-22

**Authors:** Anna Mas-Capdevila, Lisard Iglesias-Carres, Anna Arola-Arnal, Gerard Aragonès, Amaya Aleixandre, Francisca I. Bravo, Begoña Muguerza

**Affiliations:** 1 Nutrigenomics Research Group, Department of Biochemistry and Biotechnology, Universitat Rovira i Virgili, 43007 Tarragona, Spain; anna.mas@urv.cat (A.M.-C.); lisard.iglesias@urv.cat (L.I.-C.); anna.arola@urv.cat (A.A.-A.); gerard.aragones@urv.cat (G.A.); begona.muguerza@urv.cat (B.M.); 2 Department of Pharmacology, School of Medicine, Universidad Complutense de Madrid, 280040 Madrid, Spain; amaya@med.ucm.es; 3 EURECAT-Technology Centre of Catalonia, Technological Unit of Nutrition and Health, 43204 Reus, Spain

**Keywords:** bioactive peptide, endothelial dysfunction, blood pressure, bioactivity, nitric oxide, spontaneously hypertensive rats

## Abstract

AVFQHNCQE is an antihypertensive nonapeptide obtained from a chicken foot protein hydrolysate. The present study aims to investigate the mechanisms involved in its blood pressure (BP)-lowering effect. Male (17–20 weeks old) spontaneously hypertensive rats (SHR) were used in this study. Rats were divided into two groups and orally administered water or 10 mg/kg body weight (bw) AVFQHNCQE. One hour post-administration, animals of both groups were intra-peritoneally treated with 1 mL of saline or with 1 mL of saline containing 30 mg/kg bw Nω-nitro-L-arginine methyl ester (L-NAME), an inhibitor of nitric oxide (NO) synthesis, or with 1 mL of saline containing 5 mg/kg bw indomethacin, which is an inhibitor of prostacyclin synthesis (*n* = 6 per group). Systolic BP was recorded before oral administration and six hours after oral administration. In an additional experiment, SHR were administered water or 10 mg/kg bw AVFQHNCQE (*n* = 6 per group) and sacrificed six hours post-administration to study the mechanisms underlying the peptide anti-hypertensive effect. Moreover, the relaxation caused by AVFQHNCQE in isolated aortic rings from Sprague-Dawley rats was evaluated. The BP-lowering effect of the peptide was not changed after indomethacin administration but was completely abolished by L-NAME, which demonstrates that its anti-hypertensive effect is mediated by changes in endothelium-derived NO availability. In addition, AVFQHNCQE administration downregulated aortic gene expression of the vasoconstrictor factor endothelin-1 and the endothelial major free radical producer NADPH. Moreover, while no changes in plasma ACE activity were observed after its administration, liver GSH levels were higher in the peptide-treated group than in the water group, which demonstrates that AVFQHNCQE presents antioxidant properties.

## 1. Introduction

Hypertension (HTN) is a chronic elevation of systemic arterial blood pressure (BP) above certain threshold values. However, the elevation in BP is only a manifestation of a progressive disease representing an important health problem [1]. HTN is considered the most preventable risk factor for cardiovascular disease (CVD), and successful HTN prevention and treatment are key to reducing the risk of CVD [2]. Thus, the current method to treat HTN is the use of long-term drug therapy. However, the use of these drugs can result in different side effects in some patients, which leads to reduced patience compliance with the treatment, increased health care costs, and preventable fatalities [3,4]. In this context, new strategies for treating HTN based on natural products could greatly benefit hypertensive patients.

Some studies have demonstrated the anti-hypertensive properties of dietary-derived peptides [5,6,7]. These bioactive peptides are potential modulators of various regulatory processes that control BP. The renin-angiotensin-aldosterone system (RAAS) plays an important role in regulating arterial pressure [8]. In RAAS, angiotensin-converting enzyme (ACE) is a key enzyme in controlling BP and converts angiotensin I to the potent vasoconstrictor angiotensin II [8]. Thus, the inhibition of this enzyme has become an important target for drugs combating HTN. In fact, the inhibition of this enzyme is the main mechanism implied in the anti-hypertensive effect of dietary peptides [9,10,11]. In addition, endothelial function, which maintains normal vascular tone and blood fluidity to maintain normotensive BP values, has been demonstrated to be the target for some anti-hypertensive peptides [12,13,14]. In HTN, endothelial function is impaired, which presents a decreased presence of vasodilator factors, such as nitric oxide (NO) or prostacyclin (PGI_2_), and/or increased presence of endothelium-derived contracting factors, such as endothelin 1 (ET-1) [15]. In this sense, some peptides have been demonstrated to present NO-mediated anti-hypertensive effects [13,16]. NO is synthesized by the enzyme endothelial NO synthase (eNOS), which uses arginine as a substrate to produce NO. L-arginase (Arg-1), which is the enzyme that degrades arginine, has been demonstrated to play a crucial role in NO production and in the development of vascular disease [17]. In contrast, Krüpple-like factor 2 (KLF-2) has been demonstrated to be an eNOS promoter [18]. KLF-2 can also inhibit the expression of other important genes involved in regulating vessel tone, such as ET-1 [19]. KLF-2 activation is regulated by Sirtuin-1 (Sirt-1), which is also well known to activate eNOS by deacetylation and, thereby, increases the production of NO and promotes endothelial-dependent vasodilatation [20]. In addition, Sirt-1 promotes the inhibition of the activity of NADPH oxidase (NOX) [21], which is the major free radical producer in the endothelium and is known to be overexpressed in spontaneously hypertensive rats (SHR). The NOX links with the presence of endothelial dysfunction in this animal model [22]. Similarly, reactive oxygen species (ROS) have several effects on vascular and endothelial function, such as the inactivation of the vasodilator NO by O_2_^−^ [23], contributing to the development of endothelial dysfunction [15]. Some studies have demonstrated that anti-hypertensive peptides can also present antioxidant activity by enhancing one of the main endogenous antioxidant system known as reduced glutathione (GSH) [24,25].

In a previous study, the peptide sequence AVFQHNCQE was identified in the anti-hypertensive chicken foot hydrolysate Hpp11 [26]. This peptide presented in vitro ACE inhibitory (ACEI) activity and exhibited anti-hypertensive activity in SHR at a dose of 10 mg/kg body weight (bw). Nevertheless, the underlying mechanisms involved in the anti-hypertensive effect of AVFQHNCQE are still unknown. Thus, the aim of this study was to evaluate the involvement of endothelial-relaxing factors as possible anti-hypertensive mechanisms of AVFQHNCQE. We used SHR alternatively treated with Nω-nitro-L-arginine methyl ester (L-NAME), an inhibitor of NO synthesis, or with indomethacin, which is an inhibitor of PGI_2_ synthesis. Furthermore, in an additional experiment, the concentration of liver GSH, activity of plasma ACE, and aortic expression levels of different genes involved in endothelial dysfunction were evaluated in AVFQHNCQE-treated SHR.

## 2. Materials and Methods

### 2.1. Reagents

L-NAME (PubChem CID: 135193), indomethacin (PubChem CID: 3715), ACE (peptidyl-dipeptidase A, E.C. 3.4.15.1) (PubChem CID: 329770629), N-hippuryl-His-Leu (Hip-His-Leu) (PubChem CID: 94418), monochlorobimane (PubChem CID: 114886), glutathione-S transferase from horse liver (PubChem CID: 114886), acetylcholine (PubChem CID: 187), and methoxamine hydrochloride (PubChem CID: 6081) were purchased from Sigma-Aldrich (Madrid, Spain). The peptide AVFQHNCQE was synthetized by CASLO Aps (Kongens Lyngby, Denmark), and its purity of 98.94% was verified by HPLC–MS. Heparin was purchased from DeltaLab (Barcelona, Spain) (PubChem CID: 772). All other chemical solvents used were of an analytical grade.

### 2.2. Experimental Procedure in Animals

Male SHR (17–20 weeks old) weighing 298 ± 2 g were used. All animals were obtained from the Charles River Laboratories (Barcelona, Spain). The animals were maintained at a temperature of 23 °C with 12 h light/dark cycles and consumed tap water and a standard diet (STD, Panlab A04, Panlab, Barcelona, Spain) *ad libitum* during the experiments. The STD diet had energy content of 20% protein, 4% fat, 76% carbohydrates, and 0.3% Na. The animals were administered tap water or 10 mg/kg bw AVFQHNCQE dissolved in tap water by oral gavage between 8:00 and 9:00 a.m. The total volume orally administered to the rats was 1 mL in all cases. One hour after the oral administration, animals in each group (water or peptide) were intra-peritoneally administered 1 mL saline solution (water + saline or AVFQHNCQE + saline groups, respectively). The remaining rats were divided into four groups including two groups that were intra-peritoneally administered 30 mg/kg bw L-NAME (water + 30 mg/kg L-NAME or AVFQHNCQE + 30 mg/kg L-NAME) and two groups that were administered 5 mg/kg bw indomethacin (water + 5 mg/kg indomethacin or AVFQHNCQE + 5 mg/kg indomethacin groups) (*n* = 6 per group). L-NAME and indomethacin were dissolved in saline solution. Furthermore, the volume injected into rats was 1 mL in all treatment groups. Systolic blood pressure (SBP) was recorded in the rats by the tail cuff method [27] before and 6 h after water or peptide administration. To guarantee the reliability of the measurements, we established a training period of 10 days before the actual trial time, and, during this period, the rats became accustomed to the procedure. Moreover, to minimize stress-induced variations in BP, all measurements were taken by the same person in the same peaceful environment. Nevertheless, the researcher assigned to carry out the measurements did not know the exact treatment of each animal.

**Figure 1:**
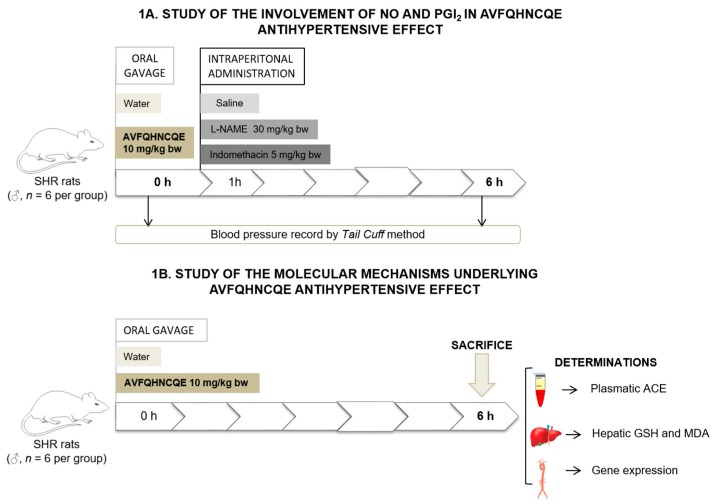
Graphical representation of the experimental design for Nω-nitro-L-arginine methyl ester hydrochloride (L-NAME) and indomethacin study in spontaneously hypertensive rats (SHR) (**1A**) and graphical representation of the experimental design used to study the effects of peptide administration on the renin-angiotensin-aldosterone system (RAAS), endothelial function, and oxidative stress in SHR (**1B**).

An additional experiment was performed using 17-week to 20-week-old male SHR weighing 302 ± 4 g. Housing and diet conditions were the same as those described in the previous experiment. Animals were administered 1 mL of tap water (*n* = 6 per group) or 10 mg/kg bw AVFQHNCQE dissolved in 1 mL of tap water (*n* = 6 per group) by oral gavage and sacrificed by live decapitation 6 h post-administration. Total blood was collected in heparin tubes. Plasma was obtained by blood centrifugation (1500× *g*, 15 min, 4 °C), and aorta and liver were excised and immediately frozen in liquid nitrogen. Both plasma and tissues were stored at −80 °C until further use. Figure 1 shows a graphical representation of both experimental designs used in this study. 

### 2.3. Determination of Plasma ACE Activity

ACE activity was measured in the plasma following the method reported by Mas-Capdevila et al. [28]. Commercial ACE at different concentrations was used to obtain a calibration curve. Plasma ACE activity was expressed (mU ACE/mL) as the mean ± standard error of the mean (SEM) of plasma for at least three replicates.

### 2.4. Reduced Glutathione Assay

The GSH assay was performed in the liver following the monochlorobimane fluorometric method [29]. GSH levels were evaluated using 90 µL of homogenized supernatant from the liver mixed with monochlorobimane (100 mM) and 10 µL of the catalyst, glutathione S-transferase solution (1 U/mL). The levels of GSH were quantified using a multi-scan microplate fluorimeter (FLUOstar optima, BMG Labtech, Offeuburg, Germany) and expressed as the mean ± SEM µmol GSH/g tissue protein for at least three replicates. Protein content was determined by the bicinchoninic acid method using the standard Pierce BCA protein assay (ThermoFisher Scientific, Madrid, Spain). The assay was conducted according to the manufacturer’s instructions in a microplate format. A calibration standard curve was prepared with seroalbumin bovine.

### 2.5. Measurement of Malondialdehyde Production

Hepatic malondialdehyde (MDA) were measured by a thiobarbituric acid assay [30] modified as described by Quiñones et al. [31]. Liver homogenates were mixed with 20 % trichloroacetic acid in 0.6 M HCl (1:1, *v*/*v*) and kept on ice for 20 min. The samples were centrifuged at 1500× *g* for 15 min before adding thiobarbituric acid (120 mM in 260 mM Tris, pH 7.0) to the supernatant in a proportion of 1:5 (*v*/*v*). The mixture was subsequently heated at 97 °C for 30 min. Spectrophotometric measurements at 540 nm were conducted at 20 °C. The liver thiobarbituric acid reactive substances (TBARS) were expressed as nmol MDA/g tissue protein. Protein content was determined by the bicinchoninic acid method using the standard Pierce BCA protein assay.

### 2.6. RNA Extraction and mRNA Quantification by Real-Time qPCR

The thoracic aorta was homogenized in TissueLyser (Qiagen, Barcelona, Spain) while RNA extraction was performed using the RNeasy Mini Kit (Qiagen). Total extracted RNA was quantified in a Nanodrop 100 Spectrophotometer (ThermoFisher Scientific). mRNA reverse transcription was carried out by using the High Capacity cDNA Reverse Transcription Kit (Applied Biosystems, Madrid, Spain). Quantitative PCR amplification and detection were performed in the CFX96 Touch Real Time PCR System (Bio-Rad, Barcelona, Spain) using 96-well plates and SYBR PCR Premix Reagent Ex Taq™ (Takara, Barcelona, Spain) following the commercial protocol.

Relative mRNA levels of eNOS, Arg-1, Klf-2, Sirt-1, NOX4, and ET-1 were analyzed by real-time PCR using glyceraldehyde 3-phosphate dehydrogenase (GADPH) as the housekeeping gene. The primers used for the different genes are shown in Table 1 and were obtained from Biomers (Söflinger, Germany). Primer specificity was verified by melting curve analysis and the amplicon size was verified by 3% agarose gel electrophoresis. The efficiency of qPCR was calculated by evaluating a 2-fold dilution series of aortic cDNA and calculated by E = 10(1/slope). The results were expressed as the logarithm of the cDNA concentration vs. the obtained Ct. The relative expression was calculated by dividing the ECt of the studied gene by the ECt of the housekeeping gene used as the control and then divided by the value of the control and water group gene expression. Each sample was performed in triplicate.

### 2.7. Experiments in Aortic Rings

Male Sprague-Dawley (SD) rats, which were 17 to 22 weeks old and weighed 240 to 305 g, were sacrificed by decapitation. To perform the experiment, the thoracic aorta was excised from each of the animals, and excess fat and connective tissue were removed. To obtain the aortic preparations, the tissue was placed in a dissecting dish containing Krebs-Henseleit solution (118 mM NaCl, 4.7 mM KCl, 2.5 mM CaCl_2_, 1.2 mM KH_2_PO_4_, 1.2 mM MgSO_4_, 25.0 mM NaHCO_3_, and 10.0 mM glucose) and cut into 3 to 4 mm rings. Aortic rings were mounted between two steel hooks in organ baths containing Krebs-Henseleit solution at 37 °C and continuously bubbled with a 95% O_2_ and 5% CO_2_ mixture, which gave a pH of 7.4. An optimal tension of 2 g was applied to all the aortic rings, and the preparations were adjusted every 15 min during the 60 to 90 min equilibration period before evaluating the tested compounds. The isometric tension was recorded by using an isometric force displacement transducer connected to an acquisition system (Protos 5, Panlab, Barcelona, Spain). Then, after the equilibration, 80 mM KCl was added to verify their functionality. When the contraction had reached the steady state (approximately 15 min after the administration), the preparations were washed to recover basal tension.

The presence of endothelium was confirmed by relaxing in response to the addition of 10 μM acetylcholine of segments previously contracted by treatment with 10^−5^ M methoxamine. A relaxation equal to or greater than 80 % was considered evidence of the functional integrity of the endothelium, and the absence of relaxation in response to acetylcholine was considered the absence of endothelium.

The rings were exposed to 10^−5^ M methoxamine to obtain steady state contraction, and AVFQHNCQE curves (10^−8^–10^−4^ M) were assayed in the methoxamine-pre-constricted rings. Water, as a negative control, was added to the bath at the same volumes used to assay the AVFQHNCQE dose-response curve. The relaxation responses were expressed as a percentage of the pre-contraction induced by methoxamine, which was considered to be 100 percent. Concentration–dependent response curves were fitted to a logistic equation, and a statistical analysis was performed to compare the curves.

All animal protocols followed in this study were approved by the Bioethical Committee of the Universitat Rovira i Virgili (reference number 8868 by Generalitat de Catalunya) (European Commission Directive 86/609) and the Spanish Royal Decree 223/1988.

### 2.8. Statistical Analysis

The results were expressed as the mean ± SEM of 6 animals per group or at least 6 experiments including the aortic rings extracted from 6 different animals in the vascular reactivity study. The data from the L-NAME and indomethacin experiments were analyzed by one-way ANOVA (Tukey’s test) using IBM SPSS Statistics 20.0 software (SPSS, Inc., Chicago, IL, USA). Plasma ACE activity, GSH content, and gene expression results were analyzed by the unpaired Student’s t test for independent samples using IBM SPSS Statistics 20.0 software (SPSS, Inc.). Differences between concentration–response curves in aortic ring experiments were analyzed by two-way analysis of variance (two-way ANOVA). Outliers were identified and eliminated by using the Grubbs’ test. Differences between groups were considered significant when *p* < 0.05.

## 3. Results

### 3.1. Effects of AVFQHNCQE on Blood Pressure in SHR Treated with L-NAME or Indomethacin

**Figure 2:**
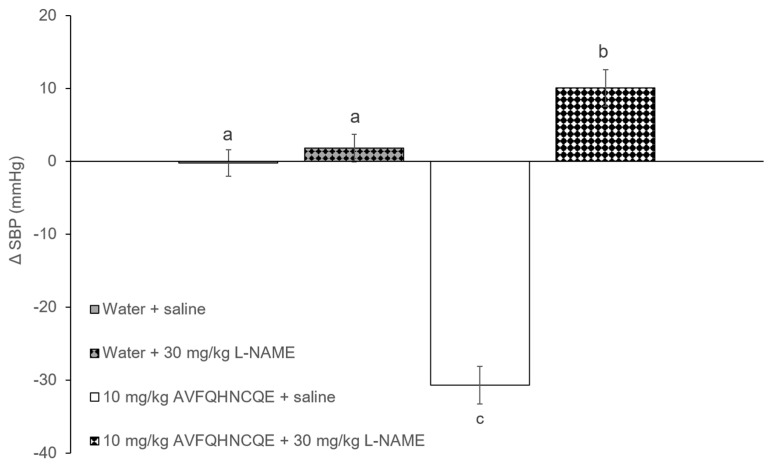
Changes in systolic blood pressure (SBP) caused in spontaneously hypertensive rats (SHR) 6 h post-administration of water or AVFQHNCQE and saline or Nω-nitro-L-arginine methyl ester hydrochloride (L-NAME). Data are expressed as the mean ± standard error (SEM) of six animals. Different letters represent statistically significant differences (*p* ≤ 0.05). The value of p was estimated by one-way ANOVA.

**Figure 3:**
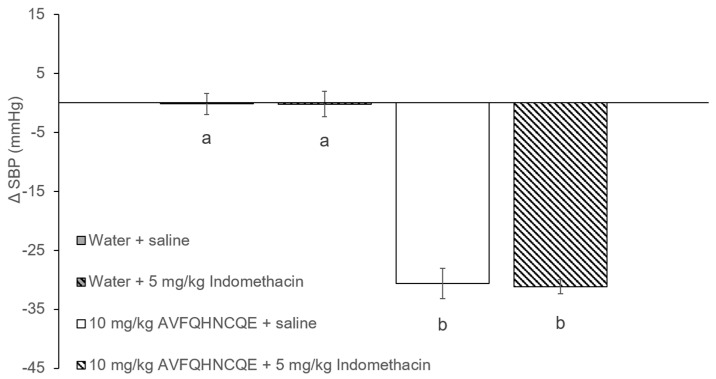
Changes in systolic blood pressure (SBP) caused in spontaneously hypertensive rats (SHR) 6 h post-administration of water or AVFQHNCQE and saline or Indomethacin. Data are expressed as the mean ± standard error (SEM) of six animals. Different letters represent statistically significant differences (*p* ≤ 0.05). The value of p was estimated by one-way ANOVA.

The initial values of SBP in the SHR were 205.2 ± 1.3 mmHg. Figure 2 and Figure 3 show the changes in SBP in SHR groups that were administered water or 10 mg/kg bw of the peptide AVFQHNCQE, which were treated intraperitoneally with saline solution, L-NAME, or indomethacin. As expected, the SHR that received water (water + saline group) did not experience changes in their SBP values 6 h after administration. However, the oral administration of 10 mg/kg bw of AVFQHNCQE (AVFQHNCQE + saline group) produced a significant decrease in SBP (-31.0 ± 2.5 mmHg, *p* ≤ 0.05) (Figure 2 and Figure 3). Nevertheless, when animals receiving the peptide were intra-peritoneally treated with L-NAME (AVFQHNCQE + 30 mg/kg of L-NAME group), the anti-hypertensive effect of this peptide disappeared and even resulted in a significant increase in SBP when compared to the SBP in the water + saline group (+ 10.1 ± 2.5 mmHg; *p* ≤ 0.05). In contrast, the anti-hypertensive effect of the peptide was also observed in animals that were also intra-peritoneally injected with 5 mg/kg bw indomethacin (AVFQHNCQE + 5 mg/kg of indomethacin group). The SBP reduction in the animals administered peptide and treated with indomethacin, and the SBP decreases in the animals administered peptide and treated with saline had nearly the same values of −31.0 ± 1.2 mmHg and −31.1 ± 1.8 mmHg, respectively.

### 3.2. Effects of AVFQHNCQE on Plasma ACE Activity, Liver GSH Concentration, Malodialdehyde Production, and Endothelial-Related Gene Expression

**Figure 4:**
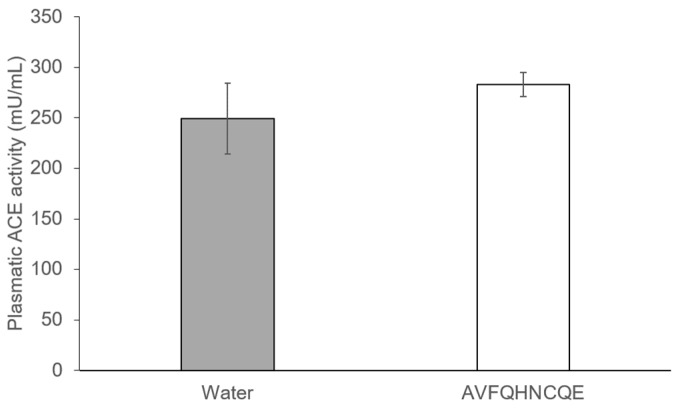
The effect of the peptide AVFQHNCQE on plasma ACE activity 6 hours after administration in spontaneously hypertensive rats. Values are means ± SEM (*n* = 6 per group). The results were analyzed with Student’s t test. The differences between the means were considered significant when *p* ≤ 0.05. No significant differences were found.

The administration of AVFQHNCQE to the SHR did not produce any effect on plasma ACE activity and similar values were found between the group administered water and the group administered peptide (Figure 4).

**Figure 5:**
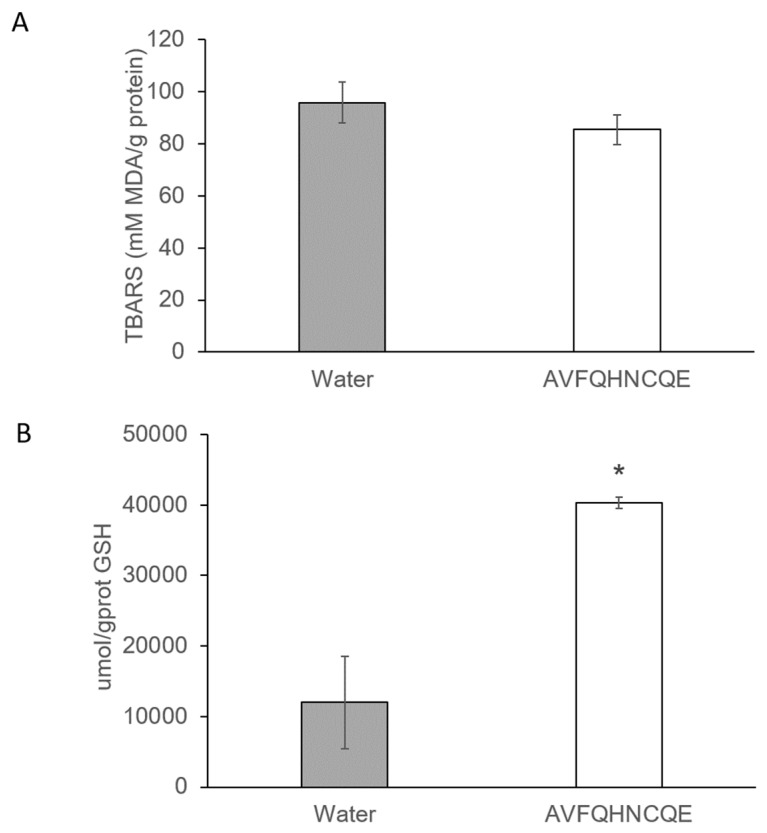
Hepatic malondialdehyde (MDA) production (**A**) and hepatic reduced gluthatione (GSH) concentration (**B**) 6 hours after administration of the peptide AVFQHNCQE in spontaneously hypertensive rats. Results are expressed as the mean ± SEM (*n* = 6 per group). The results were analyzed with the Student’s t test, and the differences between the means were considered significant when *p* ≤ 0.05. The asterisk indicates statistically significant differences.

Regarding the MDA formation, as well as the lipid peroxidation product, and GSH production in liver, no differences were found in the MDA formation between the untreated and the peptide-treated group (Figure 5A). However, after 6 hours, AVFQHNCQE administration produced a marked increase in GSH levels when compared to the group that received water (Figure 5B). Furthermore, hepatic GSH production was 70.27 % higher in the AVFQHNCQE-treated group than in the water-treated group.

**Figure 6:**
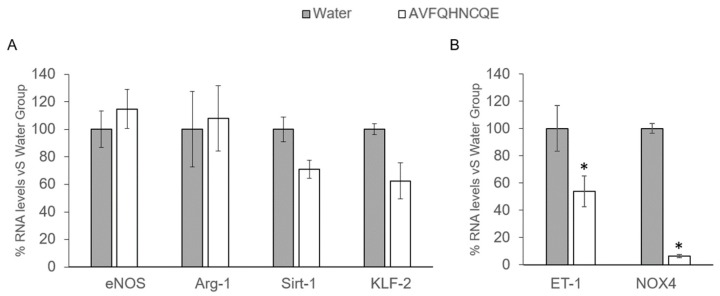
The effect of the peptide AVFQHNCQE on the expression of genes involved in the nitric oxide pathway (**A**) and on genes related to vasoconstriction and radical oxygen species production (**B**) in the endothelium 6 hours after administration in spontaneously hypertensive rats. Data are mean ± SEM (*n* = 6 per group). The results were analyzed with Student’s t test, and the differences between the means were considered significant when *p* ≤ 0.05. The asterisks indicate statistically significant differences. eNOS: endothelial nitric oxide synthase; Arg-1: L-arginase; Sirt-1: sirtuin-1; KLF-2: Krüpple-like factor 2; ET-1: endothelin 1; NOX4: NADPH oxidase 4.

Peptide administration did not produce any significant changes in the expression of the eNOS, Arg-1, KLF-2, and Sirt-1 genes (Figure 6A) but produced a significant decrease in ET-1 and NOX4 expression (Figure 6B), which shows a reduction of 46.12% and 93.59% for ET-1 and NOX4, respectively, when compared to the group administered water.

### 3.3. Effects of AVFQHNCQE on Aortic Rings

**Figure 7:**
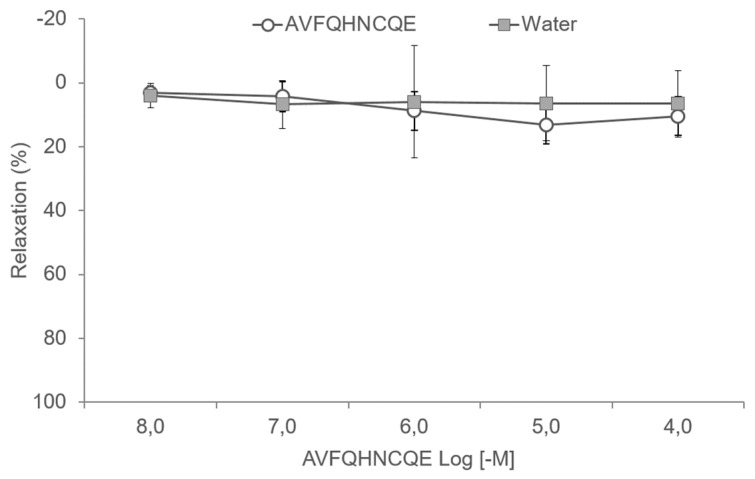
Cumulative concentration–response curves of AVFQHNCQE (10^−8^–10^−4^ M) in methoxamine pre-constricted aortic rings from Sprague-Dawley rats. Tap water was employed as a control. The same volume of water used to carry out the concentration-response curves of the peptide was added. Data are mean values ± SEM (*n* = 6 per group). No significant differences were observed between the control group and the AVFQHNCQE group.

To evaluate the effects of AVFQHNCQE on vascular reactivity, the peptide was studied in methoxamine pre-constricted aortic ring preparations. Figure 7 shows that AVFQHNCQE did not induce relaxation in aortic ring preparations from SD rats.

## 4. Discussion

Dietary proteins have been demonstrated to be a potential source of bioactive peptides and to exhibit different bioactivities, including anti-hypertensive activity [32,33]. It has been reported that some chicken proteins can yield peptides with ACEI and anti-hypertensive properties [34,35,36]. In this regard, a previous study carried out by our group in SHR [26] demonstrated that the treatment with the chicken foot-derived peptide AVFQHNCQE at a dose of 10 mg/kg bw produced a significant decrease in SBP 6 hours after oral administration. This peptide was identified from the chicken foot hydrolysate Hpp11 obtained after a chicken foot treatment (100 °C, 1.5 h) and subsequent protein hydrolysis (2 h, 50 °C, pH = 7.0) with the proteolytic enzyme Protamex [26].

Anti-hypertensive peptides mainly target ACE inhibition. However, the pathophysiology of HTN is complex, and there are other potential targets where these bioactive peptides may exert their specific anti-hypertensive actions [37]. Considering that the mechanisms involved in the anti-hypertensive effect exerted by the chicken foot-derived peptide AVFQHNCQE were unknown, this study aimed to elucidate how this peptide produces an in vivo anti-hypertensive effect. Specifically, this study evaluates the potential participation of endothelial-relaxing factors in the anti-hypertensive effect of AVFQHNCQE. Thus, to evaluate the implication of NO in the anti-hypertensive effect of the peptide, L-NAME, an inhibitor of eNOS [38], was administered to SHR. NO induces the production of cAMP and consequently increases dilatation in the blood vessels and decreases BP [39]. The results presented in this study provide clear proof of the participation of NO in the BP-lowering effect of AVFQHNCQE, as shown by the disappearance of its anti-hypertensive effect in L-NAME-treated rats. Kouguchi et al. observed similar results for a chicken collagen hydrolysate, which demonstrates that collagen-derived peptides can exhibit vasoprotective functions via NO production and effectively protect against atherogenesis [40]. Additionally, the NO-mediated anti-hypertensive effects of other anti-hypertensive peptides, including those derived from casein or whey proteins, which also exhibited antioxidant effects, were previously described [33]. In this study, despite that no significant reduction was observed in the production of hepatic MDA in the group treated with the peptide, it was demonstrated that AVFQHNCQE exhibited antioxidant effects by increasing the production of hepatic GSH, which is the main endogenous antioxidant system that reduces oxidative stress and free radical damage [41]. The measurement of the production of this molecule in the liver is a potential indication of an improvement in the oxidative stress, which was already described for SHR [42,43,44] by considering that glutathione deficiency may play a key role in the pathogenesis of many diseases including HTN [45]. These results are in concordance with other studies that revealed the presence of an antioxidant effect after the administration of bioactive peptides [46,47].

In HTN, oxidative stress limits NO bioavailability [2] and the presence and possible involvement of oxidative stress in HTN in SHR has been reported [48,49]. In fact, free radicals in the endothelium can directly scavenge NO and avoid NO-dependent vasodilatation. Thus, considering that the antihypertensive effect of the AVFQHNCQE was NO-mediated, the antioxidant effect produced by this peptide could contribute to the reduction of oxidative stress and consequently to the increase in NO bioavailability. Similarly, Dávalos et al. also reported antioxidant activity for ACEI egg white protein-derived peptides, which suggests that both mechanisms including the inhibition of ACE and the antioxidant effect could contribute to the control of HTN [24]. Considering that NO was clearly involved in the anti-hypertensive effect of AVFQHNCQE, it was evaluated whether or not the genes involved in the NO production pathway were overexpressed in the animals that received the peptide. However, no significant changes in mRNA expression of eNOS, Arg-1, Sirt-1, and KLF-2 were observed. In addition, ET-1 and NOX4 mRNA expression was evaluated after peptide administration. ET-1 is considered the main vasoconstrictor in the endothelium [50], and NOX4 is the main producer of free radicals in the vasculature [21]. In particular, an increase of NOX4 under stressful conditions was previously found to be detrimental [51]. In this study, the administration of AVFQHNCQE reduced the expression of ET-1. Previous studies have demonstrated that the modulation of endothelial ET-1 release was key in the BP-lowering effect of several other anti-hypertensive peptides, including milk-protein derived peptides [52]. In addition, Fernández-Musoles et al. reported the capacity of lactoferricin B-derived peptides, which demonstrated ACEI properties inhibit the endothelin-converting enzyme responsible for ET-1 production [53]. Moreover, AVFQHNCQE was able to reduce the expression of NOX4. A reduction in this enzyme is related to a reduction in ROS production. In fact, it has been demonstrated that a reduction of this enzyme mediates the anti-hypertensive effect of the commonly used anti-hypertensive drug atorvastatin [54]. Thus, considering these findings, an improved endothelium function contributes to the anti-hypertensive effect of AVFQHNCQE.

Considering these findings, which demonstrate that AVFQHNCQE decreases BP via NO, partly due to a reduction in the expression of NO scavengers known as ET-1 and NOX4, it was expected that this peptide would also be able to induce vasodilation in aortic ring preparations. However, the peptide did not relax the methoxamine-contracted aortic ring preparations. Thus, our in vivo and ex vivo results are not in accordance. Nevertheless, we should not forget that the aorta is a conduit artery, and the resistance arteries determine the arterial BP more than the large vessels [55]. In addition, it should be highlighted that SD rats are normotensive animals and AVFQHNCQE could present different effects in the arteries of hypertensive animals. Therefore, we believe that the vasodilation produced by NO is involved in the anti-hypertensive effect of the peptide, despite that, in this experiment, we could not demonstrate the relaxing properties of the peptide.

The endothelium also secretes other vasodilator agents than NO such as PGI_2_. Although NO is considered as the more important vasodilator in endothelium, PGI_2_ was the first endothelium-derived relaxing substance described. This prostaglandin is mainly produced by the cyclooxygenase enzyme (COX), which is also released in the endothelium and is considered a central cardioprotective hormone [56,57]. To evaluate the implication of the vasodilator PGI_2_ in the BP-lowering effect of the peptide, the anti-hypertensive effect was evaluated in rats treated with Indomethacin, which is a COX inhibitor. Although some peptides produce PGI_2_-mediated anti-hypertensive effects, such as novokinin, which is a potent anti-hypertensive peptide designed based on the structure of ovokinin [58], in this study, indomethacin treatment did not modify the anti-hypertensive effect of AVFQHNCQE. Therefore, considering this result, the participation of PGI_2_ in the anti-hypertensive effect of AVFQHNCQE could be discarded.

Lastly, considering that AVFQHNCQE has shown in vitro ACEI activity [26], the in vivo effect of this peptide on ACE activity was also studied. However, the in vitro ACEI activity of the peptide did not correspond to the same bioactivity in vivo. Thus, no changes were observed in the plasma ACE activity of peptide-treated SHR 6 hours after administration when compared to the plasma ACE activity in the water-treated SHR. Although oral administration of ACE inhibitors has been widely used to decrease BP in animal models and humans, it remains unclear which organs target these drugs to achieve their anti-hypertensive effects [59]. Moreover, in contrast to the multitude of studies that have addressed the effects of peptide application on BP, a relatively small number of studies have quantified ACEI effects of peptides in vivo [60]. In fact, ACE inhibitory peptides are required to be resistant to the gastrointestinal digestion, to be absorbed in order to reach their target organs intact, and to yield their anti-hypertensive effect [61]. Therefore, ACE inhibitory peptides are usually small peptides that are easily absorbed in an active form [33]. On the other hand, the current opinion about large anti-hypertensive peptides, such as AVFQHNCQE, is that these do not pass into the bloodstream in a significant amount and that they produce its physiological effects by interacting with receptors on the intestinal wall [62]. In this sense, it has been reported that some large peptides produce an NO-mediated anti-hypertensive effect, such as the one reported in this study, by interacting with opioid receptors in the gastrointestinal tract. This implies that their absorption is not required [63,64]. Thus, as extensively demonstrated, mechanisms other than ACE inhibition are involved in the peptides anti-hypertensive effect [14] and the physiological and pharmacological consequences of the lack of effectivity on ACE in vivo do not seem to reduce the therapeutic effect of these anti-hypertensive agents [65]. 

## 5. Conclusions

**Figure 8:**
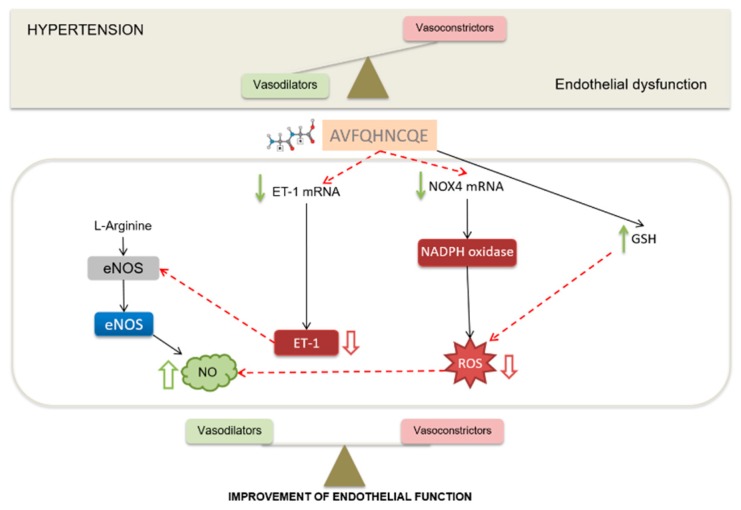
Hypertension (HTN) is characterized by the presence of endothelial dysfunction. This pathological state is defined as an imbalance between the vasodilator (mainly nitric oxide, NO) and the vasoconstriction (mainly endothelin-1, ET-1) endothelial factors. The administration of AVFQHNCQE produced an NO-mediated anti-hypertensive effect and it was expected to create an increase in NO bioavailability. NADPH oxidase 4 (NOX4) mRNA expression was found to be decreased in rats administered the peptide, which would lead to a decrease in reactive oxygen species (ROS) production and, thus, to an increase in NO availability. The mRNA expression of ET-1, vasoconstrictor, and inhibitor of NO synthesis, was also found to decrease. Lastly, the peptide increased the production of reduced glutathione (GSH), which is the main endogen antioxidant system that decreases ROS and, thus, reduces NO scavenging. All these findings lead to enhance nitric oxide, and, therefore, reduce the vasoconstriction observed under HTN conditions.

In this study with SHR, as represented in Figure 8, we demonstrated the participation of NO in the anti-hypertensive effect exerted by chicken foot-derived peptide AVFQHNCQE. This peptide could enhance NO availability by reducing oxidative stress. In addition, the reduction in ET-1 and NOX4 mRNA expression after peptide administration also contributed to improved endothelium functionality and, therefore, reduced BP. Nevertheless, since it is unknown if this peptide sequence is susceptible to gastrointestinal digestion, resulting in new peptide fragments that might be responsible for the AVFQHNCQE bioactivity, further studies to investigate the peptide digestibility, absorption, and bioavailability are in progress.

## 6. Patents

Patent application “Procedure for obtaining a hydrolysate claw chicken leg with antihypertensive activity, and peptides obtained hydrolysate containing.” ES201531372A. 11 December 2017.

## Figures and Tables

**Table 1:** List of primer characteristics.

Rat Primers	Sequence (5′……3′)	Size of Amplicon	Efficiency	GenBank Accession No.
eNOS Fw	GGATTCTGGCAAGACCGATTAC	159	2.06 (100.67%)	NM_021838.2
eNOS Rv	GGTGAGGACTTGTCCAAACACT			
Arg-1 Fw	GGCAGTGGCGTTGACCTTGT	158	2.14 (105.64%)	NM_017134.3
Arg-1 Rv	AGCAGCGTTGGCCTGGTTCT			
Sirt Fw	TTGGCACCGATCCTCGAA	217	2.02 (97.49%)	NM_001007684.1
Sirt Rv	ACAGAAACCCCAGCTCCA			
KLF2 Fw	GGACGCACACAGGTGAGAA	186	2.18 (107.85%)	NM_001007684.1
KLF2 Rv	ACATGTGTCGCTTCATGTGC			
ET-1 Fw	TGATTCTCTTGCCTCTTCTTG	110	3.08 (136.04%)	NM_012548.2
ET-1 Rv	TATGGAATCTCCTGGCTCTC			
NOX4 Fw	GTGTCTGCATGGTGGTGGTA	150	2.24 (110.62%)	NM_053524.1
NOX4 Rv	TCAACAAGCCACCCGAAACA			
GADPH Fw	CCATGTTCGTCATGGGTGTG	91	1.97 (98.93%)	NM_002046.4
GADPH Rv	GGTGCTAAGCAGTTGGTGGTG			

## References

[1] Messerli F.H., Williams B., Ritz E. (2007). Essential hypertension. Lancet.

[2] Oparil S., Acelajado M.C., Bakris G.L., Berlowitz D.R., Cífková R., Dominiczak A.F., Grassi G., Jordan J., Poulter N.R., Rodgers A. (2018). Hypertension. Nat. Rev. Dis. Primers.

[3] Reiner Ž. (2009). How to improve cardiovascular diseases prevention in Europe?. Nutr. Metab. Cardiovasc. Dis..

[4] Antonakoudis G., Poulimenos L., Kifnidis K., Zouras C., Antonakoudis H. (2007). Blood pressure control and cardiovascular risk reduction. Hippokratia.

[5] Quirós A., del Mar Contreras M., Ramos M., Amigo L., Recio I. (2009). Stability to gastrointestinal enzymes and structure–activity relationship of β-casein-peptides with antihypertensive properties. Peptides.

[6] Yamamoto N. (1997). Antihypertensive peptides derived from food proteins. Biopolym. Pept. Sci. Sect..

[7] Hong F., Ming L., Yi S., Zhanxia L., Yongquan W., Chi L. (2008). The antihypertensive effect of peptides: A novel alternative to drugs?. Peptides.

[8] Atlas S.A. (2007). The renin-angiotensin aldosterone system: Pathophysiological role and pharmacologic inhibition. J. Manag. Care Pharm..

[9] Guo Y., Pan D., Tanokura M. (2009). Optimisation of hydrolysis conditions for the production of the angiotensin-I converting enzyme (ACE) inhibitory peptides from whey protein using response surface methodology. Food Chem..

[10] Majumder K., Chakrabarti S., Morton J.S., Panahi S., Kaufman S., Davidge S.T., Wu J. (2015). Egg-derived ACE-inhibitory peptides IQW and LKP reduce blood pressure in spontaneously hypertensive rats. J. Funct. Foods.

[11] Miguel M., Manso M., Aleixandre A., Alonso M.J., Salaices M., Lopez-Fandino R. (2007). Vascular effects, angiotensin I-converting enzyme (ACE)-inhibitory activity, and anti hypertensive properties of peptides derived from egg white. J. Agric. Food Chem..

[12] Nonaka A., Nakamura T., Hirota T., Matsushita A., Asakura M., Ohki K., Kitakaze M. (2014). The milk-derived peptides Val-Pro-Pro and Ile-Pro-Pro attenuate arterial dysfunction in L-NAME-treated rats. Hypertens. Res..

[13] Tatemoto K., Takayama K., Zou M.X., Kumaki I., Zhang W., Kumano K., Fujimiya M. (2001). The novel peptide apelin lowers blood pressure via a nitric oxide-dependent mechanism. Regul. Pept..

[14] Udenigwe C.C., Mohan A. (2014). Mechanisms of food protein-derived antihypertensive peptides other than ACE inhibition. J. Funct. Foods.

[15] Hadi H.A.R., Carr C.S., Al Suwaidi J. (2005). Endothelial dysfunction: Cardiovascular risk factors, therapy, and outcome. Vasc. Health Risk Manag..

[16] Matoba N., Usui H., Fujita H., Yoshikawa M. (1999). A novel anti-hypertensive peptide derived from ovalbumin induces nitric oxide-mediated vasorelaxation in an isolated SHR mesenteric artery. FEBS Lett..

[17] Durante W., Johnson F.K., Johnson R.A. (2007). Arginase: A critical regulator of nitric oxide synthesis and vascular function. Clin. Exp. Pharmacol. Physiol..

[18] Pons Z., Margalef M., Bravo F.I., Arola-Arnal A., Muguerza B. (2016). Grape seed flavanols decrease blood pressure via Sirt-1 and confer a vasoprotective pattern in rats. J. Funct. Foods.

[19] Atkins G.B., Jain M.K. (2007). Role of Krüppel-Like Transcription Factors in Endothelial Biology. Circ. Res..

[20] Martínez-Fernández L., Pons Z., Margalef M., Arola-Arnal A., Muguerza B. (2014). Regulation of Vascular Endothelial Genes by Dietary Flavonoids: Structure-Expression Relationship Studies and the Role of the Transcription Factor KLF-2. J. Nutr. Biochem..

[21] Zarzuelo M.J., López-Sepúlveda R., Sánchez M., Romero M., Gómez-Guzmán M., Ungvary Z., Pérez-Vizcaíno F., Jiménez R., Duarte J. (2013). SIRT1 inhibits NADPH oxidase activation and protects endothelial function in the rat aorta: Implications for vascular aging. Biochem. Pharmacol..

[22] Zalba G., Beaumont F.J., José G.S., Fortuño A., María A., Etayo J.C., Díez J., Jose G.S., Fortun A., Dı J. (2000). Vascular NADH/NADPH Oxidase Is Involved in Enhanced Superoxide Production in Spontaneously Hypertensive Rats. Hypertension.

[23] Cai H., Harrison D.G. (2000). Endothelial dysfunction in cardiovascular diseases: The role of oxidant stress. Circ. Res..

[24] Udenigwe C.C., Udechukwu M.C., Yiridoe C., Gibson A., Gong M. (2016). Antioxidant mechanism of potato protein hydrolysates against in vitro oxidation of reduced glutathione. J. Funct. Foods.

[25] He R., Alashi A., Malomo S.A., Girgih A.T., Chao D., Ju X., Aluko R.E. (2013). Antihypertensive and free radical scavenging properties of enzymatic rapeseed protein hydrolysates. Food Chem..

[26] Bravo F.I., Arola L., Muguerza B. (2017). Procedure for Obtaining a Hydrolyzate Claw Chicken Leg with Antihypertensive Activity, and Peptides Obtained Hydrolyzate Containing. ES Patent.

[27] Buñag R.D., Butterfield J. (1982). Tail-cuff blood pressure measurement without external preheating in awake rats. Hypertension.

[28] Miguel M., Alonso M.J., Salaices M., Aleixandre A., López-Fandiño R. (2007). Antihypertensive, ACE-inhibitory and vasodilator properties of an egg white hydrolysate: Effect of a simulated intestinal digestion. Food Chem..

[29] Kamencic H., Lyon A., Paterson P.G., Juurlink B.H.J. (2000). Monochlorobimane Fluorometric Method to Measure. Anal. Biochem..

[30] Rodriguez-Martinez M.A., Ruiz-Torres A. (1992). Homeostasis between lipid peroxidation and antioxidant enzyme activities in healthy human aging. Mech. Ageing Dev..

[31] Quiñones M., Guerrero L., Suarez M., Pons Z., Aleixandre A., Arola L., Muguerza B. (2013). Low-molecular procyanidin rich grape seed extract exerts antihypertensive effect in males spontaneously hypertensive rats. Food Res. Int..

[32] Martínez-Maqueda D., Miralles B., Recio I., Hernández-Ledesma B. (2012). Antihypertensive peptides from food proteins: A review. Food Funct..

[33] Hernández-Ledesma B., Del Mar Contreras M., Recio I. (2011). Antihypertensive peptides: Production, bioavailability and incorporation into foods. Adv. Colloid Interface Sci..

[34] Yuliatmo R., Fitriyanto N.A., Bachruddin Z., Erwanto Y. (2017). Increasing of angiotensin converting enzyme inhibitory derived from chicken leg. Int. Food Res. J..

[35] Sangsawad P., Roytrakul S., Yongsawatdigul J. (2017). Angiotensin converting enzyme (ACE) inhibitory peptides derived from the simulated in vitro gastrointestinal digestion of cooked chicken breast. J. Funct. Foods.

[36] Terashima M., Baba T., Ikemoto N., Katayama M., Morimoto T., Matsumura S. (2010). Novel angiotensin-converting enzyme (ACE) inhibitory peptides derived from boneless chicken leg meat. J. Agric. Food Chem..

[37] Majumder K., Wu J. (2015). Molecular Targets of Antihypertensive Peptides: Understanding the Mechanisms of Action Based on the Pathophysiology of Hypertension. Int. J. Mol. Sci..

[38] Moncada S., Higgs E.A. (1991). Endogenous nitric oxide: Physiology, pathology and clinical relevance. Eur. J. Clin. Investig..

[39] Loscalzo J., Welch G. (1995). Nitric oxide and its role in the cardiovascular system. Prog. Cardiovasc. Dis..

[40] Kouguchi T., Zhang Y., Sato M., Takahata Y., Morimatsu F., Parthasarathy S. (2012). Vasoprotective Effect of Foods as Treatments: Chicken Collagen Hydrolysate. Atherogenesis.

[41] Elias R.J., Kellerby S.S., Decker E.A. (2008). Antioxidant activity of proteins and peptides. Crit. Rev. Food Sci. Nutr..

[42] Wind S., Beuerlein K., Armitage M.E., Taye A., Kumar A.H.S., Janowitz D., Neff C., Shah A.M., Wingler K., Schmidt H.H.H.W. (2010). Oxidative stress and endothelial dysfunction in aortas of aged spontaneously hypertensive rats by NOX1/2 is reversed by NADPH oxidase inhibition. Hypertension.

[43] Jin L., Piao Z.H., Sun S., Liu B., Kim G.R., Seok Y.M., Lin M.Q., Ryu Y., Choi S.Y., Kee H.J. (2017). Gallic Acid Reduces Blood Pressure and Attenuates Oxidative Stress and Cardiac Hypertrophy in Spontaneously Hypertensive Rats. Sci. Rep..

[44] Manso M.A., Miguel M., Even J., Hernández R., Aleixandre A., López-Fandiño R. (2008). Effect of the long-term intake of an egg white hydrolysate on the oxidative status and blood lipid profile of spontaneously hypertensive rats. Food Chem..

[45] Wu G., Fang Y.-Z., Yang S., Lupton J.R., Turner N.D. (2004). Glutathione metabolism and its implications for health. J. Nutr..

[46] Shimizu S., Shimizu T., Tsounapi P., Higashi Y., Martin D.T., Nakamura K., Honda M., Inoue K., Saito M. (2015). Effect of silodosin, an alpha1A-adrenoceptor antagonist, on ventral prostatic hyperplasia in the spontaneously hypertensive rat. PLoS ONE.

[47] Sohaib M., Anjum F.M., Sahar A., Arshad M.S., Rahman U.U., Imran A., Hussain S. (2017). Antioxidant proteins and peptides to enhance the oxidative stability of meat and meat products: A comprehensive review. Int. J. Food Prop..

[48] Suzuki H., Swei A., Zweifach B.W., Schmid-Schönbein G.W. (1995). In vivo evidence for microvascular oxidative stress in spontaneously hypertensive rats. Hydroethidine microfluorography. Hypertension.

[49] Negishi H., Njelekela M., Ikeda K., Sagara M., Noguchi T., Kuga S., Kanda T., Liu L., Nara Y., Tagami M. (2000). Assessment of in vivo oxidative stress in hypertensive rats and hypertensive subjects in Tanzania, Africa. Hypertens. Res..

[50] Masaki T., Sawamura T. (2006). Endothelin and endothelial dysfunction. Proc. Jpn. Acad. Ser. B.

[51] Konior A., Schramm A., Czesnikiewicz-Guzik M., Guzik T.J. (2014). NADPH Oxidases in Vascular Pathology. Antioxid. Redox Signal..

[52] Maes W., Van Camp J., Vermeirssen V., Hemeryck M., Ketelslegers J.M., Schrezenmeir J., Van Oostveldt P., Huyghebaert A. (2004). Influence of the lactokinin Ala-Leu-Pro-Met-His-Ile-Arg (ALPMHIR) on the release of endothelin-1 by endothelial cells. Regul. Pept..

[53] Fernández-Musoles R., López-Díez J.J., Torregrosa G., Vallés S., Alborch E., Manzanares P., Salom J.B. (2010). Lactoferricin B-derived peptides with inhibitory effects on ECE-dependent vasoconstriction. Peptides.

[54] Cui W., Matsuno K., Iwata K., Ibi M., Katsuyama M., Kakehi T., Sasaki M., Ikami K., Zhu K., Yabe-Nishimura C. (2009). NADPH Oxidase Isoforms and Anti-hypertensive Effects of Atorvastatin Demonstrated in Two Animal Models. J. Pharmacol. Sci..

[55] Pons Z., Arola L. (2013). Involvement of nitric oxide and prostacyclin in the antihypertensive effect of low-molecular-weight procyanidin rich grape seed extract in male spontaneously hypertensive rats. J. Funct. Foods.

[56] Mitchell J.A., Ali F., Bailey L., Moreno L., Harrington L.S. (2008). Role of nitric oxide and prostacyclin as vasoactive hormones released by the endothelium. Exp. Physiol..

[57] Moncada S., Gryglewski R., Bunting S., Vane J.R. (1976). An enzyme isolated from arteries transforms prostaglandin endoperoxides to an unstable substance that inhibits platelet aggregation. Nature.

[58] Yamada Y., Yamauchi D., Usui H., Zhao H., Yokoo M., Ohinata K., Iwai M., Horiuchi M., Yoshikawa M. (2008). Hypotensive activity of novokinin, a potent analogue of ovokinin(2–7), is mediated by angiotensin AT2 receptor and prostaglandin IP receptor. Peptides.

[59] Takai S., Jin D., Sakaguchi M., Miyazaki M. (2004). Significant target organs for hypertension and cardiac hypertrophy by angiotensin-converting enzyme inhibitors. Hypertens. Res..

[60] Martin M., Deussen A. (2017). Effects of natural peptides from food proteins on angiotensin converting enzyme activity and hypertension. Crit. Rev. Food Sci. Nutr..

[61] Mine Y., Shahidi F. (2006). Nutraceutical Proteins and Peptides in Health and Disease.

[62] Miner-Williams W.M., Stevens B.R., Moughan P.J. (2014). Are intact peptides absorbed from the healthy gut in the adult human?. Nutr. Res. Rev..

[63] Nurminen M.L., Sipola M., Kaarto H., Pihlanto-Leppälä A., Piilola K., Korpela R., Tossavainen O., Korhonen H., Vapaatalo H. (2000). Alpha-lactorphin lowers blood pressure measured by radiotelemetry in normotensive and spontaneously hypertensive rats. Life Sci..

[64] Sipola M., Finckenberg P., Vapaatalo H., Pihlanto-Leppälä A., Korhonen H., Korpela R., Nurminen M.-L. (2002). Alpha-lactorphin and beta-lactorphin improve arterial function in spontaneously hypertensive rats. Life Sci..

[65] Costerousse O., Allegrini J., Clozel J.P., Ménard J., Alhenc-Gelas F. (1998). Angiotensin I-converting enzyme inhibition but not angiotensin II suppression alters angiotensin I-converting enzyme gene expression in vessels and epithelia. J. Pharmacol. Exp. Ther..

